# Surgical Care of Major Newborn Malformations

**Published:** 2013-01-01

**Authors:** Yogesh K Sarin

**Affiliations:** Department of Pediatric Surgery, Maulana Azad Medical College, New Delhi - 110002, India

 Focusing on the surgical care of major newborn malformations, this book published in November 2012 by World Scientific Publishing Co. Pvt. Ltd. from Singapore, encompassing 386 pages and 16 chapters, emphasizes clinical management and diagnosis, reviews operative techniques and situates the present approach of management to these patients in historical context to encourage ongoing advancement in the care of the these patients. This volume addresses the major “index cases” involving neonates that are taught in pediatric surgical training programs. The editors/ authors have successfully facilitated to help clinicians sculpt a creative and adaptable surgical approach that can be individualized for each child.

The opening chapter on “perioperative management of the neonatal patient” covers physiological considerations, preoperative care, operative and postoperative care. Sections on informed consent, bowel preparation, invasive monitoring through umbilical vessels, total parenteral nutrition and pain management have been beautifully elucidated. A special mention about management of very small for gestational age and preterm neonates is commendable.

While discussing the malrotation, the author has laid emphasis on the current changed focus of radiologists away from the vessels and towards the retroperitoneum; ultrasonic confirmation that the duodenum lies retro-mesenteric crossing the midline excludes malrotation. The authors have also sensitized the readers with the role of computed tomography scans in malrotation and midgut volvulus. Further, while role of both open and laparoscopic methods have been explained, it is well highlighted that laparoscopy is not suitable for unstable patient or one with presumed intestinal necrosis.

The next few chapters on bowel atresia, Hirschsprung’s disease, anorectal malformations, abdominal wall defects, congenital diaphragmatic hernia, extra hepatic biliary atresia are well illustrated and exhaustive. Ovarian cysts and sacrococcygeal teratomas have also been touched upon.

Meconium syndromes used as an umbrella term for wide range of pathologies- meconium ileus, meconium plug syndrome, small left colon syndrome, meconium obstruction in very small premature neonate and meconium peritonitis is novel.

The chapter on necrotizing enterocolitis (NEC) presents the debate on primary peritoneal drainage versus laparotomy in the management of these patients. In the complication section, the authors have additionally highlighted the non-gastrointestinal complications such as impaired growth rates and neurodevelopmental delay in the survivors of the disease. The role of probiotics in the prevention of NEC is also mentioned.

Before reading here in the chapter on esophageal atresia, I was not aware that the said entity was featured in the commercial hit movie “M*A*S*H”. The resident doctors would find comprehensive details of surgical technique and also mention of different techniques that could be done for pure esophageal atresia.

My personal pick of the book is chapter on malformations of the lung. I must admit that I was unaware of some terminologies such as congenital pulmonary airway malformation (CPAM) replacing congenital cystic adenomatoid malformation (CCAM), and congenital lobar overinflation (CLO) replacing congenital lobar emphysema (CLE). From antenatal therapy to minimally invasive surgery, all is lucidly described with help of algorithm, table and illustrations.

Last but not the least, the chapter on vascular and lymphatic anomalies is an academic treat. Supported by 30 illustrations, this chapter covers everything about vascular tumors to vascular malformations including complex-combined vascular malformations including CLOVES Syndrome (did I hear -what is that?).

A must-have textbook on neonatal surgery, which has finally filed the slot vacated by the good old Lister and Irving’s book on Neonatal Surgery {1} that adorned the library shelves way back in 1980-90s. I strongly recommend you buy one from www.amazon.com for $82.89.

## Footnotes

**Source of Support:** Nil

**Conflict of Interest:** Author belongs to editorial board however he was not involved in evaluation and decision making about the manuscript.

